# Effects of a 6-month dual-task, power-based exercise program on cognitive function, neurological and inflammatory markers in older adults: secondary analysis of a cluster randomised controlled trial

**DOI:** 10.1007/s11357-024-01316-8

**Published:** 2024-08-28

**Authors:** Jamie L. Tait, Rachel L. Duckham, Timo Rantalainen, Catherine M. Milte, Luana C. Main, Caryl A. Nowson, Kerrie M. Sanders, Dennis R. Taaffe, Keith D. Hill, Gavin Abbott, Robin M. Daly

**Affiliations:** 1 Institute for Physical Activity and Nutrition (IPAN), School of Exercise and Nutrition Sciences, Deakin University, Geelong, VIC Australia; 2 Australian Institute for Musculoskeletal Science (AIMSS), Department of Medicine-Western Health, the University of Melbourne, Melbourne, VIC Australia; 3 Faculty of Sport and Health Sciences and Gerontology Research Center, University of Jyväskylä, Jyväskylä, Finland; 4 Department of Medicine, Western Health, the University of Melbourne, Melbourne, VIC Australia; 5 Exercise Medicine Research Institute and School of Medical and Health Sciences, Edith Cowan University, Joondalup, WA Australia; 6 Rehabilitation Ageing and Independent Living (RAIL) Research Centre, Monash University, Melbourne, VIC Australia

**Keywords:** Dual-task training, Cognition, Ageing, Cytokine, Physical activity

## Abstract

Functional power-based exercise training can improve physical performance in older adults and cognitive training can improve measures of cognition, but their combined effects on cognition and related risk factors (neurological and inflammatory markers) remains uncertain. This 6-month cluster randomised controlled trial evaluated the effectiveness of dual-task functional power training (DT-FPT) on cognition and circulating neurological and inflammatory markers in older adults at increased falls risk, and whether intervention responses varied by apolipoprotein-E (ApoE) and brain derived neurotrophic factor (BDNF) polymorphisms. Three hundred residents aged ≥ 65 years at increased falls risk residing in 22 independent-living retirement communities, were randomised by village, to DT-FPT (n = 156, 11 villages) involving a multi-component power-based training program performed simultaneously with cognitive and/or motor tasks (45–60 min, 2/week), or a usual care control (CON) group (n = 144, 11 villages). Cognition (computerized CogState battery), inflammatory cytokines, BDNF, insulin-like growth factor-1, vascular endothelial growth factor, amyloid β (1–40) and (1–42) were assessed at baseline and 6-months. Overall, 233 (78%) participants completed the intervention and adherence averaged 50.1%. DT-FPT led to a net 0.18–0.20 SD benefit versus CON in psychomotor ability/attention and reaction time/attention (both P < 0.05). There were no significant intervention effects on circulating markers, except for a net 10.5% benefit in amyloid β (1–40) in DT-FPT versus CON (P < 0.05). Responses were not influenced by APOE or BDNF genotype. In conclusion, DT-FPT in older adults at increased falls risk can provide some cognitive benefits, but these were not related to corresponding changes in inflammatory or neurological markers or influenced by genotype. Australian New Zealand Clinical Trials Registry (ACTRN12613001161718). http://www.anzctr.org.au/ This project was funded by a grant from the National Health and Medical Research Council (NHMRC) Project (APP1046267).

**Supplementary Information:**

The online version contains supplementary material available at 10.1007/s11357-024-01316-8.

## Introduction

Age-associated cognitive decline and dementia pose significant public health challenges for which there is no cure. Regular exercise, including aerobic training, progressive resistance training (PRT) or the combination, can elicit cognitive benefits in healthy older adults and those with cognitive impairment [1]. Cognitive training, involving mental exercises like remembering word lists and visual tasks, can also improve cognition in older adults however intervention findings are mixed [2]. Combined exercise and cognitive/motor training may be more effective than exercise alone [3, 4], however the effectiveness of dual-task simultaneous training, where exercise is performed concurrently with a secondary cognitive and/or motor task, remains uncertain, with reported cognitive benefits possibly dependent on the mode of exercise, and the type of cognitive and motor training involved [5, 6]. A meta-analysis of studies investigating simultaneous exercise-cognitive training reported some cognitive benefits with various modalities including aerobic training, PRT, functional (stepping) training, or the combination, and exergaming [7]. High velocity-PRT, or power training is another form of training that is effective at improving muscle power and functional performance in older adults [8], and can enhance cognitive abilities in frail elderly [9], and cognitively impaired older adults [10]. Integrating challenging balance training as part of a multimodal exercise-cognitive program can also enhance cognitive function [7]. Despite these benefits, no studies to our knowledge have evaluated the effectiveness of integrating power and challenging balance training as part of a multi-component program, which includes functional and mobility movements resembling daily activities (e.g., stepping, squatting) performed with a rapid concentric phase, all completed simultaneously with cognitive-motor training, on cognitive function in healthy older adults.

It is plausible that exercise-induced improvements in inflammatory and neurological mediators [e.g., brain derived neurotrophic factor (BDNF) and insulin-like growth factor] associated with cognition and various chronic diseases, or alterations to plasma levels of amyloid beta (Aβ) (1–42) and (1–40), may underlie cognitive and physical benefits following dual-task simultaneous exercise-cognitive training [11, 12]. Aerobic training, PRT, or multimodal training can reduce circulating inflammatory cytokines in older adults with and without various chronic disease(s) [13, 14]. However, studies to date that have included power training [15] and circuit-based functional training [16] have reported minimal changes, which may relate to relatively short intervention durations (i.e., 12 weeks), the recruitment of physically active healthy older participants within a restricted age range (i.e., 60–75 years), inclusion of women only or relatively low training volumes (i.e. 20 min per session). In healthy older adults and those with chronic disease, multimodal training that includes PRT [17], and cognitive training independently [18], have been shown to increase BDNF levels, which plays a key role in neuronal survival and growth [19]. However, there is currently limited and inconclusive evidence as to whether combined exercise-cognitive training can beneficially modify circulating inflammatory and/or neurological markers [20–22]. Furthermore, particular genetic polymorphisms (e.g., Met allele on gene encoding BDNF; APOE-ε4 variant coding for the apolipoprotein E protein) may influence the effect of exercise on the release of these biomarkers and cognitive performance [23, 24], but this has not been examined in dual-task exercise-cognitive studies. Therefore, the aim of this study was to assess whether a 6-month, dual-task multi-component functional power training (DT-FPT) program could improve cognitive function, and circulating levels of neurological and inflammatory markers, compared to usual care in older adults at increased falls risk living independently in retirement villages. A secondary aim was to determine whether the response of cognition and biomarkers to the intervention varied by genetic factors (presence of a Met allele within the *BDNF* gene and/or ε4 variant of the *APOE* gene).

## Methods

### Study design

This study is a secondary analysis from an 18-month community-based, cluster randomised controlled trial (RCT) in which older adults at increased falls risk residing in independent living retirement villages were randomized (by village) by an independent researcher to receive: 1) a multi-component, functional dual-task power training program with challenging balance/mobility training that was performed simultaneously with a secondary attention-demanding motor or cognitive task (DT-FPT, n = 11 villages, n = 156 participants), or 2) a usual care control group (CON, n = 11 villages, n = 144 participants). The primary aim of the 18-month RCT, which was divided into three 6-month phases (phase 1: supervised/structured; phase 2: maintenance period; phase 3: follow-up), was to evaluate the effectiveness of the intervention on falls rate and any sustained benefits [25]. This pre-planned study examined secondary outcomes after the 6-month supervised and structured intervention, including cognitive function and additional inflammatory and neurological markers which were introduced after the publication of the study protocol [25]. Recruitment occurred in three cohorts over a 14-month period from February 2014 to April 2015 [26], with cohorts 1, 2 and 3 completing the 6-month intervention in January 2015, June 2015 and December 2015, respectively. Eight retirement villages (82 participants) were recruited into cohort 1, six villages (104 participants) into cohort 2 and eight villages (114 participants) into cohort 3. Baseline and follow-up testing at 6-months were conducted at each retirement village. Cluster randomisation was employed by village to avoid the likelihood of intervention contamination within each village site, and group assignment was performed by a researcher not involved in the study [25]. All testing personnel were blinded to the group allocation, except for one researcher (JT) responsible for participant communication and administering cognitive tests. The study was approved by Deakin University Human Research Ethics Committee (EC 2013–051) and registered on the Australian and New Zealand Clinical Trials Registry (ACTRN12613001161718).

### Participants

A total of 300 older adults (81 men and 219 women) from 22 retirement villages in the greater metropolitan and regional areas of Melbourne, Australia were recruited into the trial. Recruitment and screening details were previously published [25]. Briefly, interested participants were screened over the phone and included if aged 65 years and over, scored ≥ 3 on a defined algorithm adapted from prior research on falls risk factors [27], made no more than two errors on the Short Portable Mental Screening Questionnaire (to exclude cognitive impairment), spoke English proficiently and walked unaided or with minimal assistance for at least 50 m. Eligible participants were further screened with the Exercise and Sports Science Australia exercise-screening tool. Participants answering ‘Yes’ to any survey questions required medical clearance from their physician prior to participating in the intervention. Participants were ineligible based on: 1) current or prior participation in ≥ 150 min of moderate-vigorous physical activity per week or participation in structured PRT or balance programs more than once weekly in the past 3 months; 2) musculoskeletal or neurological diseases or acute/terminal illness that may limit training; 3) unstable cardiovascular or respiratory disorders; 4) upper or lower limb fracture in past 3 months, or 5) visual impairment not corrected with glasses/lenses. Written informed consent was obtained before commencing the study.

### Intervention

Villages with participants allocated to the exercise intervention were prescribed two 45—60 min supervised, group-based (8—10 per group) sessions per week on non-consecutive days for 6 months, led by trained exercise professionals on-site at each village. Each training session involved four components: 1) a warm-up consisting of rhythmic and range of motion exercises (e.g., marching, rolling shoulders); 2) 2—3 challenging balance and mobility exercises which simulated common daily functional tasks (e.g., seated centre of gravity exercises – leaning/trunk rotation/leg extensions; marching, side stepping, multidirectional weight shifts, standing on uneven surface; walking over obstacle courses); 3) high-velocity functional power training (FPT) consisting of 5—6 lower leg exercises (e.g., squats, lunges, leg extensions, hip abduction/adduction), at least one upper limb exercise (e.g. bent-over rows, wall push-up) and core stability/postural exercises (e.g., fitball sit-ups, back extensions); and 4) cool-down. Participants completed 2 sets of 10—20 repetitions for stepping exercises progressing to 50 repetitions. For FPT, 2 sets of 10—15 reps with a target of 4 to 6 (moderate to hard) on the 10-point Borg Rating of Perceived Exertion scale [28]. Programs were individually progressed based on participant improvement and perceived exertion. Dual-task training components were performed simultaneously with the challenging balance/mobility and FPT exercises, and involved a combination of cognitive (e.g., solving anagrams), visual (e.g., memory recall) and motor tasks (e.g., throwing a weighted ball). Example exercises included step-ups while reciting lists, dynamic lunges while solving anagrams, and tandem walking on a beam while throwing a medicine ball to a partner. The 6-month program, as reported previously [25], comprised a 2-week familiarisation phase and three 8-week blocks, each with smaller microcyles for program and exercise progression. Training was implemented and supervised by Accredited Exercise Physiologists and experienced Certificate III/IV personal trainers who had all completed a ‘train-the-trainer’ education session run by the research team to ensure consistent program delivery across villages.

### Control group

Participants in villages allocated to the usual care control group maintained their normal physical activity habits, and received usual care from their medical practitioner and community services. Participants were given a falls prevention booklet (Department of Health: ‘Don’t fall for it – falls can be prevented’, and ‘An active way to better health’) and encouraged to achieve the recommended 150 min of weekly physical activity through pamphlets and initial contact with the research team.

### Cognitive measures

Domains of cognitive function were assessed through CogState computerized cognitive battery (CogState Ltd, Melbourne, Australia) using the following tests: Groton Maze Learning Test (GMT), which measures executive function, memory, and visuospatial learning; Detection task (DET): simple reaction time and processing speed; Identification task (IDN): choice reaction time and visual attention; One Card Learning task (OCL): attention and visual memory; and the One Back task (ONB): working memory and attention. CogState is valid and reliable across various ages and clinical groups and can be completed with limited computer experience, making it suitable for older adults [29]. The battery was administered on a laptop and completed under researcher guidance. Participants received standardised verbal instructions and a practice test before each task. Outcome measures for these tasks were reaction time (ms) of correct responses (IDN, DET, ONB), proportion of correct responses (OCL), and total number of errors (GMT). As previously reported [30], the reaction time scores of IDN, DET and ONB were log_10_ transformed, while the square root of the proportion of correct responses on the OCL task was arcsine transformed. Raw scores at baseline and 6-months for each task were then transformed into a Z-score using the mean and standard deviation (SD) at baseline of the total sample in the study. From these individual Z-scores, composites were created for the following: global cognitive function (GCF: average for GMT, DET, IDN, OCL, and ONB), Learning-Working memory (L-WM: average for OCL and ONB), Psychomotor function-Attention (Psy-Att: average for DET and IDN), and the CogState Brief Battery (CBB: average for DET, IDN, OCL and ONB). Higher scores on these composites indicate better performance [30]. Participants with a Z-score ≤ -1.0 SD on at least three of the five individual cognitive tests were classified as having mild cognitive impairment (MCI) (based on communication with CogState Pty Ltd and [29]).

### Inflammatory, neurological and hormonal markers

Fasted, morning (8-10am) venous blood samples were drawn at baseline and 6-months. All biomarkers were analysed in duplicate. Serum interleukin (IL)-4, IL-6, IL-1β, IL-8, tumor necrosis factor (TNF)-α and IL-10 were measured using the Milliplex T Cell high-sensitivity human cytokine panel (Merck Millipore, Australia). All analyses recorded an intra-assay % coefficient of variation (%CV) of < 11% and an inter-assay %CV of < 16%. High-sensitive CRP was quantified by Latex immunoassay (CRP Vario CRP16, Abbott Diagnostics, USA, ARCHITECT c8000) (inter-assay %CV: 2.50–5.03%). Inflammatory markers below the detection threshold were assigned the value for the assay’s lowest detectable limit. Serum concentrations of BDNF and vascular endothelial growth factor (VEGF) were determined using a commercially available ELISA kit (Duo Kit ELISA; R & D Systems, Minneapolis, USA). Serum IGF-1 concentrations were determined by a chemiluminescence immunoassay method using a Liaison IGF-1 reagent (DiaSorin S.p.A., Italy) and Liaison XL analyser. The detection limit for IGF-1 by this method was 3 ng/mL (inter-assay %CV: 0.89–2.30%). Plasma levels of Aβ (1–40) and Aβ (1–42) were measured by the assay for plasma INNO-BIA Aβ forms (Fujirebio, Europe). Concentrations below the detection threshold were assigned the value for the assay’s lowest detectable limit. Serum IGF-1 and hs-CRP were analysed at Royal Melbourne Hospital (Australia), other markers were analysed by Cardinal Bio-research Pty Ltd (Australia). Concentrations of inflammatory cytokines were standardised to a Z-score based on the sample mean and pooled SD. From these Z-scores, composites consisting of pro- (IL-6, IL-1β, IL-8, TNF-α, CRP) and anti-inflammatory cytokines (IL-4 and IL-10) were calculated (31), with higher Z-scores representing a greater circulating pro- or anti-inflammatory state, respectively. A composite inflammatory Z-score was formed by summing individual Z-scores for all seven biomarkers, with pro-inflammatory Z-scores considered positive and anti-inflammatory Z-scores as negative [31]. A higher composite Z-score indicated increased inflammation. The Aβ (1–42): Aβ (1–40) ratio was calculated by dividing the plasma values of Aβ (1–42) by Aβ (1–40). Analyses for inflammatory and neurological measures included only participants with complete covariate data (n = 268). Of the 233 participants who completed the intervention, 211 participants had complete data [DT-FPT, n = 108 (69%); CON, 103 (72%)]. Incomplete data were due to: withdrawal/loss to follow up [DT-FPT, n = 33 (21%); CON, 19 (13%)], failure to provide any sample [DT-FPT, n = 2 (1%); CON, 2 (1%)], missed baseline but provided 6-month sample [DT-FPT, n = 5 (3%); CON, 10 (7%)], and missed 6-month collection due to illness [DT-FPT, n = 8 (5%); CON, 10 (7%)].

### Genotyping

Genotype-specific cognitive and biomarker responses to the intervention were assessed using *BDNF* and *APOE* polymorphism data obtained from baseline or 6-month venous blood samples. Single nucleotide polymorphisms were genotyped as described by the manufacturer (Agena Bioscience, San Diego, USA) for BDNF (rs6265), ApoE-ε3 (rs7412) and ApoE-ε4 (rs429358) using matrix-assisted laser desorption/ionization time-of-flight mass spectroscopy (MALDI-TOF MS) (Agena Bioscience, San Diego, USA). Participants were classified based on *APOE* gene status, either having at least one ε4 allele or none, and by *BDNF* genotype, either having at least one Met allele or being Val/Val homozygotes.

### Anthropometry and body composition

Height was measured to the nearest 0.1 cm and body weight was measured to the nearest 0.01 kg using standard techniques. Body mass index (BMI) was calculated according to the equation [weight (kg) divided by height (m^2^)]. Estimates of whole-body fat percentage, fat mass (in kg) and fat-free mass (FFM, in kg) were assessed using bioelectrical impedance (TANITA BC-418, Tanita, Japan). Participants were free to continue with their usual diet during the study, with no dietary recommendations, specific food choices or regulations of diet imposed on study participants during the study period. However, participants were instructed to refrain from eating 1–2 h prior to testing and to maintain normal hydration.

### Health and medical history, and medication use

Participant health and medical history (present or past diagnosis of health condition from a list of 33 conditions), current medication use, employment status, ethnicity (based on birthplace and parent’s ethnic origin), education level, and smoking status, were assessed by questionnaire. Participants were deemed at cardiometabolic risk if they satisfied at least one of the following: taking antihypertensive medication/diagnosis of hypertension, taking lipid-lowering medication, type 2 diabetic, history of heart attack, heart disease, or angina.

### Depression Anxiety and Stress Scale (DASS-21)

The Depression Anxiety and Stress Scale (DASS)-21 assessed symptoms of depression, anxiety and stress over the past week [32]. The sum of the depression subscale was used as a covariate in assessing cognitive function.

### Adverse events

Participants in both groups were interviewed at 3- and 6-months by an unblinded researcher, to identify adverse events related to DT-FPT beyond usual care. Adverse events were defined as any unintended health-related issues (sign, symptom, syndrome, illness) that occurred or worsened during the trial. Trainers also recorded any adverse events sustained during and reported after each exercise session (e.g., illness or injury).

### Exercise adherence

Exercise program adherence was determined by tracking attendance at the supervised sessions, based on the participants’ completed exercise cards. Adherence was calculated by dividing the attended sessions by the total prescribed sessions over 6 months and multiplying by 100.

### Statistical analyses

The sample size for this study was based on the project’s primary aim to reduce the rate of falls [25]. Briefly, 118 participants per group (2-tailed, P < 0.05 and power of 0.8) were required to detect a 40% reduction in the rate of falls, allowing for a 15% dropout. To account for cluster randomisation, a conservative intra-cluster correlation coefficient of 0.01 was assumed [33], giving a design effect of 1.19, assuming approximately 15—20 participants were recruited per village. An original sample size of 280 participants from ~ 15 villages was therefore required, however due to a higher-than-expected attrition rate in Cohort 1 this number was increased to 300. This sample size from the larger study also provided > 80% power (2-tailed, P < 0.05) to detect a modest difference [effect size 0.5 (Cohen’s d)] in cognitive measures for the current study (145 required to have complete data, accounting for 15% attrition rate). Primary statistical analyses were conducted on an intention-to-treat basis and additional sensitivity analyses were conducted, using STATA release 17.0 (STATA, College Station, TX, USA). Data were checked for normality before analysis; log transformation was applied to all biomarkers except BDNF and IGF-1. An outlier was excluded from IL-4, IL-8, and CRP data due to being > 15 standard deviations above the mean, likely attributed to measurement error. The effect of the intervention on the primary (cognition) and secondary (biomarkers) outcomes for this study were analysed using linear mixed models with random effects, adjusting for the variability between clusters (villages). The models included fixed effects of group (DT-FPT or CON), time, and their interaction (which was the estimated intervention effect) and random intercepts for individuals and villages. Intervention effects on cognitive function were initially assessed without adjustment (model 1), before adjusting for age, sex, education, depression and BMI at baseline, cardiometabolic status, smoking history, BDNF Met- and ApoE-ε4 carrier status, and baseline values for the outcome measure (model 2). Intervention effects on biomarkers were assessed without adjustment (model 1), and adjusting for age, sex, cardiometabolic status and BMI at baseline, and baseline values (model 2). A per-protocol analysis was also conducted including DT-FPT participants with ≥ 50% adherence to the exercise program. An adherence of ≥ 80% was initially prespecified for the primary outcome, but because of the modest number that achieved this level (n = 36, 23%) we modified the protocol to include those with ≥ 50% adherence (equivalent to one session per week). Thus, these findings must be considered exploratory. To determine if genotype influenced the response of cognition and biomarkers to the intervention, interactions between ApoE-ε4 and BDNF Met-carrier status by group and time were independently conducted using linear mixed models. A sensitivity analysis was conducted to compare cognition between participants who completed the intervention and those who did not attend 6-month testing. Finally, multiple imputation using chained equations was used to impute missing data at baseline and follow-up, and analyses repeated for all outcomes [34]. Percentage changes for inflammatory and neurological markers (excluding BDNF and IGF-1) were derived from log-transformed within-group and between-group differences, then multiplied by 100. All data are presented as means ± SD or 95% CI. Statistical significance was set at P < 0.05 and no adjustments were made for multiple tests or co-primary outcomes. However, mean estimates and 95% confidence intervals of intervention effects are reported so that readers can make their own judgements of the relative importance of findings.

## Results

### Participant characteristics

Three hundred men and women aged 65 to 96 years (mean SD; 77.4 ± 6.8) were recruited across 22 retirement communities. One DT-FPT participant withdrew after baseline, requesting data removal, hence the final number was 299 (n = 155 for DT-FPT; n = 144 for CON). The average size of each cluster (village) was 14 participants (range 5 to 23; Online Resource 1). A total of 287 participants (96%) possessed at least one chronic health condition at baseline, 119 (40%) were classified as obese (BMI > 30; Table 1) and 43% were ex/current smokers (10 were current smokers). At baseline nearly half (49%) of the participants were taking lipid-lowering medication, and 71% were on antihypertensive medication (Table 1). No significant between-group differences were observed in medication usage changes (type, dose, or number) after 6 months. There were no significant changes in the number of diseases (total or new), or the proportion of participants with diseases after 6 months. At baseline, 22 (7%) of the participants were classified as having MCI based on the CogState tests, with equal distribution between the groups [DT-FPT: 11 (7%); CON 10: (7%)].

**Table 1:** Baseline characteristics of participants randomised to the dual task functional power training group (DT-FPT) and the usual care control (CON) group

Characteristics	DT-FPT	CON
n	155	144
Number of clusters (retirement communities)	11	11
Median number of participants per cluster	14.0 ± 8.0	11.0 ± 9.0
Women, n (%)	101 (65%)	118 (82%)
Age, years	77.2 ± 6.6	77.7 ± 7.2
Height, cm	163.2 ± 9.1	160.0 ± 8.0
Weight, kg	77.7 ± 16.0	74.3 ± 14.1
BMI (kg/m^2^)	29.1 ± 5.2	29.0 ± 4.9
Smoking status, n (%)		
* Current/ Ex-Smoker*	72 (46%)	56 (39%)
* Non-smoker*	82 (53%)	86 (60%)
DASS-21-Depression	2.0 ± 6.0	2.0 ± 6.0
ApoE-ε4 carrier, n (%)	42 (29%)	39 (28%)
BDNF Met carrier, n (%)	50 (34%)	42 (31%)
Presence of chronic health conditions*	150 (97%)	137 (96%)
* No. of conditions in those with a condition*	3.0 ± 2.0	3.0 ± 2.0
Presence of cardiometabolic risk factors, n (%)	136 (88%)	118 (82%)
Employment Status, n (%)		
* Retired / Not employed*	145 (94%)	133 (94%)
* Part time employment*	4 (3%)	3 (2%)
* Home duties / Other*	5 (3%)	6 (4%)
Caucasian, n (%)	151 (98%)	139 (98%)
Education, n (%)		
* Primary/Some High School*	57 (37%)	57 (40%)
* Completed High School/ Technical Trade Cert*	50 (32%)	49 (34%)
* University/Tertiary level*	47 (30%)	36 (25%)
Medications taken*		
* Antihypertensive, n (%)*	112 (72%)	101 (70%)
* Lipid-lowering, n (%)*	82 (53%)	63 (44%)
* NSAIDs, n (%)*	18 (12%)	22 (15%)
* Anti-depressant, n (%)*	37 (24%)	31 (22%)
* Diabetic, n (%)*	16 (10%)	18 (13%)
* Neurological, n (%)*	4 (3%)	4 (3%)
* Analgesics (non-NSAIDs), n (%)*	17 (11%)	16 (11%)
* Sleep aids, n (%)*	11 (7%)	14 (10%)

Values are mean ± SD except number of participants per cluster, DASS-21-Depression subscale score and Number of health conditions (median ± Interquartile Range). ApoE: apolipoprotein; BDNF: brain derived neurotrophic factor; BMI: body mass index; DASS: Depression, anxiety and stress scale. NSAID: Nonsteroidal anti-inflammatory drug. Neurological medication included anti-Parkinson and anti-epileptic medication. * Missing data for DT-FPT: n = 1 (1%), CON: n = 2 (1%)

### Study attrition

**Fig. 1:**
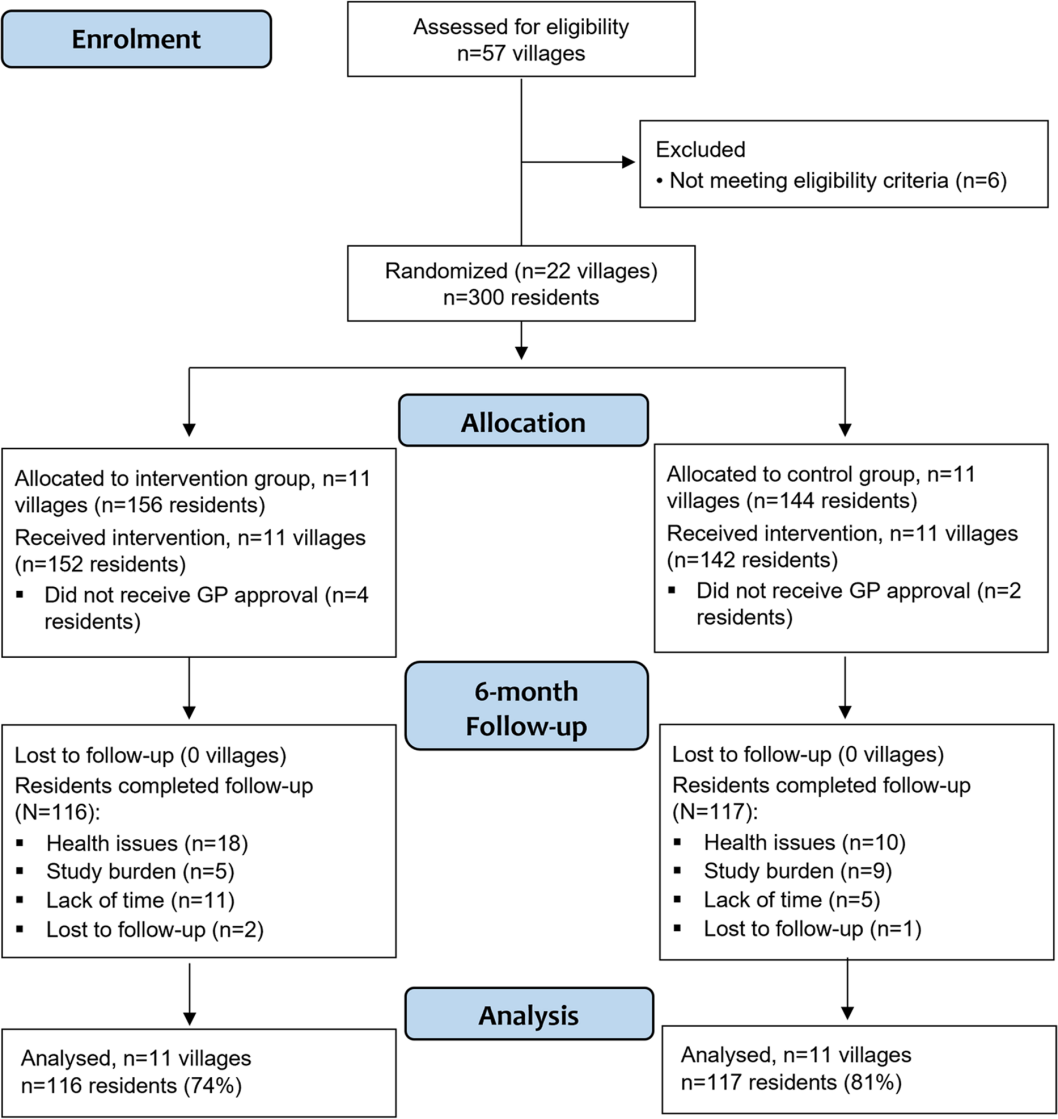
Study flow diagram of villages and participants over the 6-month intervention. NB: all analyses were conducted using an intention-to-treat approach, however the numbers for ‘analysis’ relate to participants that completed the intervention for complete-case analysis

In total, 67 (22%) participants [DT-FPT, n = 40 (25%); CON, n = 27 (19%)] did not complete 6-month follow-up testing. Of these, 49 participants withdrew from the trial [DT-FPT n = 31 (10%); CON n = 18 (6%)] with 18 of these participants unable to complete follow-up testing due to ill health (n = 15) or lost to follow-up (n = 3). Reasons for withdrawal or lack of follow-up are presented in Fig. 1 and Online Resource 1. Participants who completed the intervention had significantly higher baseline scores in all cognitive measures, except for One Card Learning, compared to those who withdrew/did not attend testing. Characteristics of these groups are presented in Online Resource 2.

### Exercise adherence and adverse events

Mean (± SD) adherence to the exercise program was 50 ± 32% (median 58%, range 0 to 98%), equivalent to approximately one session per week. Overall, 14 musculoskeletal and health complaints were reported by 12 of the 155 participants in the DT-FPT group throughout the 6-month intervention which included: joint pain (knee, shoulder, back, foot), dizziness, bronchitis and muscle strains, but no participants stopped the program or withdrew from the study due to their injury. Two of the 14 events were pre-existing injuries aggravated by the exercise program. Overall, four (33%) of the events were deemed to be related to the exercise program, seven (47%) were possibly related and three (20%) were unrelated. Seven events were resolved without sequel, four persisted without requiring treatment, and three required some form of treatment or follow-up (e.g., icing, medication, health professional review). Trainers successfully modified exercise programs for the 12 participants who reported an injury or complaint.

### Cognitive function

After 6 months, there was a statistically significant mean 0.20 SD (95% CI: 0.01, 0.39) net benefit for the change in choice reaction time/visual attention (IDN) scores and a significant net 0.18 SD (95% CI: 0.01, 0.36) benefit for the change in the composite psychomotor function-attention score in DT-FPT compared to CON (group-by-time interaction, P = 0.009 and P = 0.031, respectively) (Table 2). Both results remained significant after covariate adjustment (model 2). For other cognitive measures, there were no within group changes nor between-group differences, except that processing speed/reaction time (DET) Z-scores decreased by 0.20 SD in DT-FPT (P = 0.030) and 0.36 SD in CON (P < 0.001), while in CON there were improvements in visual learning [OCL, 0.20 SD (0.01, 0.38), P = 0.039] and composite learning-working memory scores [0.14 SD (0.04, 0.25), P = 0.007]. Similar results were observed for the per-protocol analyses, but the reported between-group differences in favour of DT-FPT were attenuated for choice reaction time/visual attention (IDN) (P = 0.122) and composite psychomotor function-attention score (P = 0.075) following data imputation (Online Resource 3). No significant interactions between *BDNF* or *APOE* genotype and changes in cognitive measures were detected.

**Table 2:** Mean baseline cognitive performance Z-Scores, the within-group changes relative to baseline and net between-group differences for the change after 6 months in the dual-task functional power training (DT-FPT) and control (CON) groups

	DT-FPT		CON		Intervention	
	n	Mean ± SD or (95% CI)	n	Mean ± SD or (95% CI)	Net Difference (95% CI)	P- values ^1^
GMT (Executive function)						
Baseline	151	0.15 ± 0.95	138	-0.17 ± 1.03		
∆ 6 months	113	0.01 (-0.13, 0.16)	113	0.05 (-0.10, 0.20)	-0.04 (-0.25, 0.16)	0.667 | 0.591
DET (Psychomotor function)						
Baseline	154	0.02 ± 1.02	144	-0.02 ± 0.98		
∆ 6 months	115	-0.20 (-0.37, -0.02)*	117	-0.36 (-0.54, -0.19)†	0.16 (-0.08, 0.41)	0.140 | 0.121
IDN (Attention/Choice reaction time)						
Baseline	154	-0.01 ± 1.01	144	0.01 ± 1.00		
∆ 6 months	115	0.08 (-0.05, 0.21)	117	-0.12 (-0.25, 0.02)	0.20 (0.01, 0.39)	0.009 | **0.025**
OCL (Visual learning)						
Baseline	153	0.08 ± 1.00	144	-0.09 ± 0.99		
∆ 6 months	114	0.07 (-0.10, 0.25)	117	0.20 (0.01, 0.38)*	-0.13 (-0.38, 0.13)	0.745 | 0.435
ONB (Working memory)						
Baseline	154	0.09 ± 1.05	144	-0.09 ± 0.94		
∆ 6 months	115	0.07 (-0.04, 0.18)	117	0.09 (-0.03, 0.22)	-0.03 (-0.19, 0.14)	0.717 | 0.910
Global cognitive function						
Baseline	151	0.08 ± 0.69	138	-0.05 ± 0.62		
∆ 6 months	113	0.004 (-0.07, 0.08)	113	-0.03 (-0.10, 0.04)	0.03 (-0.07, 0.14)	0.148 | 0.102
Learning-Working Memory						
Baseline	153	0.09 ± 0.83	144	-0.09 ± 0.73		
∆ 6 months	114	0.07 (-0.04, 0.18)	117	0.14 (0.04, 0.25)**	-0.08 (-0.22, 0.07)	0.948 | 0.840
Psychomotor function-Attention						
Baseline	154	0.003 ± 0.89	144	-0.003 ± 0.88		
∆ 6 months	115	-0.06 (-0.18, 0.06)	117	-0.24 (-0.37, -0.11)†	0.18 (0.01, 0.36)	0.031 | **0.033**
CogState Brief Battery						
Baseline	153	0.05 ± 0.74	144	-0.05 ± 0.69		
∆ 6 months	114	0.01 (-0.08, 0.09)	117	-0.05 (-0.13, 0.03)	0.06 (-0.06, 0.17)	0.085 | 0.063

Baseline values are means ± SD; within and between-group (net) differences are unadjusted means with 95% CI. Net differences (95% CIs) were calculated by subtracting within-group changes from baseline for the CON group from within-group changes for the DT-FPT group after 6 months. Missing data points for baseline due to: unable to complete cognitive test (max DT-FPT, n = 2; CON, n = 5), or failed requirements of task (max DT-FPT, n = 2; CON, n = 1). Missing data points at 6-month testing were due to failing requirements of cognitive test (DT-FPT, n = 2; CON, n = 2) and unable to complete cognitive test (DT-FPT, n = 1; CON, n = 2). ^1^ P-values for time and group-by-time interaction terms were derived from linear mixed models and represent Model 1 (unadjusted) and Model 2 (adjusted for age, sex, education level, cardiometabolic status, baseline DASS-21 depression subscale score at baseline, *APOE* genotype and *BDNF* genotype, smoking history, baseline values). DET: Detection task; GMT: Groton Maze Learning Test; IDN: Identification task; OCL: One Card Learning task; ONB: One Back task. * P < 0.05, ** P < 0.01, † P ≤ 0.001, within-group change relative to baseline

### Inflammatory markers

For all inflammatory measures and composite scores, there were no significant between-group differences (Table 3). However, in DT-FPT there was a mean 30.3% increase in serum IL-4 (P = 0.005), and a 7.3% decrease in TNF-α (P = 0.021). In CON, there were significant decreases in serum IL-10 (-16.4%, P = 0.006), IL-8 (-8.8%, P = 0.004), and the pro-inflammatory composite score (-0.10 SD, P = 0.026). All results remained relatively unchanged in per-protocol analysis and following data imputation (Online Resource 4).

**Table 3:** Mean baseline inflammatory concentrations, the within-group changes relative to baseline and net between-group differences for the change after 6 months in the dual-task functional power training (DT-FPT) and control (CON) groups

	DT-FPT		CON		Intervention effects	
	n	Mean ± SD or (95% CI)	n	Mean ± SD or (95% CI)	Net Difference (95% CI)	P- values ^1^
IL-6						
Baseline (pg/mL)	140	1.85 ± 1.67	128	3.06 ± 3.61		
% ∆ 6 months	108	5.3 (-7.7, 18.3)	103	-5.2 (-21.6, 11.1)	10.6 (-10.1, 31.3)	0.922 | 0.977
TNF-α						
Baseline (pg/mL)	140	10.2 ± 5.89	128	12.4 ± 8.68		
% ∆ 6 months	108	-7.3 (-13.6, -1.1)*	103	1.1 (-8.0, 10.1)	-8.4 (-19.2, 2.4)	0.302 | 0.262
IL-1β						
Baseline (pg/mL)	140	1.54 ± 4.25	128	1.95 ± 3.31		
% ∆ 6 months	108	-8.0 (-21.5, 5.5)	103	-1.2 (-14.0, 11.5)	-6.8 (-25.2, 11.6)	0.182 | 0.279
IL-8						
Baseline (pg/mL)	140	13.9 ± 9.08	127	17.1 ± 15.5		
% ∆ 6 months	108	-3.2 (-9.1, 2.8)	102	-8.8 (-14.6, -2.9)**	5.6 (-2.7, 13.9)	0.509 | 0.622
CRP						
Baseline (mg/L)	137	2.76 ± 3.42	126	3.37 ± 3.57		
% ∆ 6 months	106	17.9 (-0.4, 36.1)	99	4.2 (-8.4, 16.9)	13.6 (-8.7, 35.9)	0.469 | 0.473
IL-10						
Baseline (pg/mL)	140	4.65 ± 8.31	128	9.28 ± 16.8		
% ∆ 6 months	108	-5.3 (-19.1, 8.6)	103	-16.4 (-28.1, -4.8)**	11.1 (-7.0, 29.2)	0.561 | 0.623
IL-4						
Baseline (pg/mL)	140	19.2 ± 38.5	127	32.9 ± 66.3		
% ∆ 6 months	108	30.3 (9.3, 51.2)**	102	-7.3 (-25.8, 11.1)	37.6 (9.7, 65.5)	0.658 | 0.569
Pro-inflammatory Z-Score						
Baseline	140	-0.12 ± 0.51	128	0.14 ± 0.71		
∆ 6 months	108	0.06 (-0.04, 0.15)	103	-0.10 (-0.19, -0.01)*	0.16 (0.03, 0.28)	0.848 | 0.862
Anti-inflammatory Z-Score						
Baseline	140	-0.14 ± 0.57	128	0.16 ± 1.16		
∆ 6 months	108	0.02 (-0.03, 0.07)	103	-0.02 (-0.09, 0.06)	0.04 (-0.05, 0.12)	0.449 | 0.402
Single composite inflammatory Z-score						
Baseline	140	0.02 ± 0.50	128	-0.02 ± 0.91		
∆ 6 months	108	0.04 (-0.05, 0.12)	103	-0.08 (-0.18, 0.02)	0.12 (-0.02, 0.25)	0.619 | 0.619

Baseline values are means ± SD; within and between-group (net) differences are means with 95% confidence intervals (CI), and calculated by subtracting within-group changes from baseline for the CON group from within-group changes for the DT-FPT group after 6 months. Missing data points at baseline due to outliers (n = 3) and insufficient sample for CRP (n = 4). Missing data points at 6-month testing were due to insufficient sample (DT-FPT, n = 2; CON, n = 2) and outliers (CON, n = 2). Percentages are calculated as log-transformed within-group and between-group changes × 100. Inflammatory Z-scores and changes are presented as raw data, all analyses were conducted on Z scores constructed from log-transformed concentrations. ^1^ P-values were derived from linear mixed models and represent Model 1 (unadjusted) and Model 2 (adjusted for age, sex, BMI, cardiometabolic disease risk and baseline levels). CRP: C-reactive protein; IL-1β: interleukin-1 beta; IL-4: interleukin-4; IL-6: interleukin-6; IL-8: interleukin-8; IL-10: interleukin-10; TNF-α: tumor-necrosis factor alpha. * P < 0.05, ** P < 0.01

### Neurological markers

For Aβ (1–40), there was a mean 10.5% significant net benefit (decrease) for the change in DT-FPT compared to CON (group-by-time interaction, P = 0.032), due to no change in DT-FPT (-2.7%, P = 0.200) and a 7.8% (P = 0.006) increase in CON (Table 4). There were no other significant within-group or between-group differences, except that Aβ (1–42) increased by 17.8% in DT-FPT (P = 0.049). Results were unchanged in per-protocol analysis; however, differences were attenuated after multiple imputation (Online Resource 5). No significant *BDNF* or *APOE* genotype interactions were detected for any biomarker changes.

**Table 4:** Mean baseline neurological concentrations, the within-group changes relative to baseline and net between-group differences for the change after 6 months in the dual-task functional power training (DT-FPT) and control (CON) groups

	DT-FPT		CON		Intervention effects	
	n	Mean ± SD or (95% CI)	n	Mean ± SD or (95% CI)	Net Difference (95% CI)	P- values ^1^
Aβ (1–40)						
Baseline (pg/mL)	140	112.8 ± 50.4	128	109.2 ± 32.8		
% ∆ 6 months	108	-2.7 (-8.1, 2.6)	102	7.8 (2.3, 13.2)**	-10.5 (-18.1, -2.9)	**0.033** | **0.032**
Aβ (1–42)						
Baseline (pg/mL)	140	5.68 ± 6.91	128	4.27 ± 4.92		
% ∆ 6 months	108	17.8 (0.02, 35.7)*	102	13.6 (-6.9, 34.0)	4.3 (-22.6, 31.2)	0.600 | 0.610
Aβ (1–42): Aβ (1–40) ratio						
Baseline	140	0.06 ± 0.11	128	0.04 ± 0.04		
∆ 6 months	108	0.04 (-0.004, 0.07)	102	0.02 (-0.02, 0.06)	0.01 (-0.04, 0.07)	0.413 | 0.417
BDNF						
Baseline (ng/mL)	140	32.6 ± 9.20	128	28.9 ± 9.80		
% ∆ 6 months	108	-0.08 (-5.5, 5.4)	103	12.4 (0.04, 24.8)	-12.5 (-25.7, 0.74)	0.774 | 0.531
IGF-1						
Baseline (nmol/L)	137	16.9 ± 5.13	127	16.5 ± 5.08		
% ∆ 6 months	106	1.9 (-1.9, 5.7)	100	-0.2 (-3.2, 2.8)	2.1 (-2.8, 6.9)	0.434 | 0.614
VEGF						
Baseline (pg/mL)	140	341.3 ± 258.4	128	415.6 ± 401.5		
% ∆ 6 months	108	-6.1 (-15.3, 3.1)	103	-9.8 (-21.4, 1.8)	3.6 (-11.0, 18.3)	0.816 | 0.517

Baseline values are means ± SD; within and between-group (net) differences are means with 95% confidence intervals (CI), and calculated by subtracting within-group changes from baseline for the CON group from within-group changes for the DT-FPT group after 6 months. Missing data points at baseline due to insufficient sample for IGF-1 (n = 4). Missing data points at 6-month testing were due to insufficient sample (DT-FPT, n = 2; CON, n = 4). Percentages are calculated as log-transformed within-group and between-group changes × 100, except for BDNF and IGF-1 which were not transformed. BDNF and IGF-1 are presented as percentage change for consistency, however all analyses were conducted on raw concentrations. ^1^ P-values were derived from linear mixed models and represent Model 1 (unadjusted) and Model 2 (adjusted for age, sex, BMI, cardiometabolic disease risk, baseline levels). Aβ: amyloid beta; BDNF: brain derived neurotrophic factor; IGF-1: insulin-like growth factor-1; VEGF: vascular endothelial growth factor. * P = 0.05, ** P < 0.01

### Body composition

Following the intervention, there was a trend for a 0.26 kg/m^2^ net benefit (decrease) for the change in BMI in DT-FPT compared to CON (group-by-time interaction, P = 0.069) which was due to no change in DT-FPT [-0.06 (-0.21, 0.09), P = 0.271], and a 0.21 kg/m^2^ [(0.05, 0.36), P = 0.011] increase in CON (Online Resource 6). No other significant within group or between-group differences were observed for weight, fat mass, percentage body fat or FFM, except for a 0.43 kg (0.05, 0.80) increase in weight in CON (P = 0.027).

## Discussion

The main finding from this 6-month cluster RCT in older adults at increased falls risk residing in independent living retirement villages was that a twice-weekly group-based dual-task, multi-component functional power-based training program (DT-FPT) was associated with significant improvements in choice reaction time and attention, and maintenance in psychomotor ability and attention, compared to a usual care control group. However, there were no marked effects of the intervention on global cognitive function, executive function, working memory or learning. Furthermore, DT-FPT was not associated with any consistent improvements in various inflammatory and neurological biomarkers, with the exception of a significant reduction in amyloid β (1–40) relative to controls. Finally, the DT-FPT benefits to cognitive function and biomarkers were not influenced by BDNF-Met or ApoE-ɛ4 polymorphisms.

The finding that our DT-FPT intervention led to some cognitive benefits (choice reaction time, psychomotor ability and attention) in older adults is consistent with previous meta-analyses of intervention trials reporting that dual-task exercise-cognitive training in older adults can improve cognitive domains including global cognitive function, executive function, processing speed, attention, and working memory [5, 7]. Our positive findings for specific cognitive domains are likely related to the multi-task exercises included in our program, which required a distribution of attention between movement components (e.g., walking on a beam, monitoring technique whilst stepping and power training), and visual, cognitive and motor secondary tasks which engaged executive abilities encompassing aspects of attention (e.g., alerting and orienting, selective and visual attention). Consistent with these findings, several 12-week trials in community dwelling older adults involving either low-intensity PRT and walking [21], or PRT, aerobic, balance and coordination and dance [35], both performed in accordance to changing auditory cues, and combined with cognitive activities (e.g., arithmetic and word games), reported significant improvements in subscales of attention, compared to exercise training alone [21], and within group improvements in visual attention [35]. Despite the different exercise programs prescribed in these studies, using secondary tasks that require sustained or divided attention appears to be beneficial for enhancing attention in older adults.

The benefits to psychomotor abilities in our study could be attributed to the prescription of visual cognitive dual-tasks requiring quick target detection (e.g., spotting differences between two pictures) and engaging higher-order processing abilities, and secondary motor tasks focusing on the synchronisation of movement to music or visual cues (e.g., walking in time to music, catching and quickly throwing a ball to a partner). Limited data suggests that performing functional tasks may be innately cognitively demanding, providing an enriched environment for cognitive and functional improvement in both MCI, and healthy older adults [36]. In part support of our findings, a 12-week intervention in community-dwelling older adults that performed motor tasks and movement sequences as quickly as possible, concurrently with moderate-intensity aerobic training and PRT (60 min, twice weekly), displayed greater improvements in simple and choice reaction time compared to an exercise-only and a control group [37]. In contrast, a 12-week dual-task exercise program focusing on muscle strength rather than functional performance did not improve reaction time/processing speed [21]. Furthermore, previous studies have attributed benefits of cognitive training alone to the strengthening of specific brain circuits in older adults [38], while cognitive-exercise training may minimise cortical activation in the prefrontal cortex during cognitive tasks [39]. Collectively, this may enhance the efficiency of cognitive processing and reduce cognitive load and fatigue, contributing to better cognitive performance. Therefore, our observed benefits may result from the use of secondary tasks and functional training that challenged proprioception, reaction time, and/or information processing. However, further investigation into the neural correlates associated with these benefits is warranted.

We observed no effects of DT-FPT on executive function, global cognition or working memory, despite including secondary tasks that aimed to activate executive abilities and higher-order processing [e.g., working memory (remembering lists) and cognitive flexibility (anagrams)]. It is possible that the low-intensity modality of our training program may explain these findings, as exercise intensity and duration may moderate some of the cognitive benefits following combined exercise-cognitive programs [6]. Moreover, improvements in working memory and executive function have been observed in older adults engaging in moderate to high intensity aerobic training, PRT, or the combination, performed for 45–60 min per session across a range of frequencies [40]. The DT-FPT sessions in our study could be considered low-moderate intensity given that the focus was on challenging balance/mobility activities and high-velocity (power) functional movements. Furthermore, our training loads were manipulated through weighted vests, therabands, and relatively light-moderate (0.5 to 10 kg) hand-held and ankle weights. Therefore, it is possible that our training dose and intensity was insufficient for stimulating neurobiological mechanisms (e.g., increased neurogenesis, plasticity, hemodynamic activity) associated with exercise-induced benefits for these domains.

Circulating levels of IGF-1, BDNF and VEGF may mediate the relationship between exercise and cognition [11], however we observed no intervention effects in these markers. The lack of intervention effects on BDNF and IGF-1 may stem from homeostatic mechanisms that minimise growth factor fluctuations. Transient spikes in BDNF concentrations have been demonstrated after acute exercise bouts, returning to, or lower than, pre-bout concentrations within 15 to 60 min post-exercise [41]. Recent evidence also suggests that low-moderate intensity aerobic and resistance exercise training does not produce a difference in peripheral BDNF concentration [42]. In our study, blood samples were obtained within a week of the last exercise session and may therefore reflect chronic adaptations. Previous research has also suggested that exercise-induced IGF-1 released from the liver and other tissues (e.g., muscle) may be quickly sequestered by target tissue in an effort to maintain circulating homeostatic balance [43]. The relatively healthy nature of our cohort may have also created a reduced capacity for improvement in neurological markers. Increases in IGF-1 have been observed following exercise in participants with lower baseline IGF-1 levels [44], while increased BDNF levels are typically reported in older adults with comorbidities [45]. Older adults with suppressed levels of neurological markers may therefore have greater capacity for exercise-induced improvements. Additionally, ageing diminishes the release of growth factors like BDNF and IGF-1, impacting various physiological processes and functions. This decline is compounded by a reduced responsiveness of tissues to these factors. Therefore, the lack of IGF-1 response to our training program may also stem from an age-related decline in functioning of the growth hormone (GH)/IGF-1 axis, leading to reductions in GH and IGF-1 and increases in IGF binding protein, which inhibits IGF-1 action [46]. In addition, chronic inflammation associated with aging can suppress the GH/IGF-1 axis [47], further blunting IGF-1 responses to exercise. Collectively, our data does not currently support a stimulating effect of dual-task exercise on circulating BDNF, IGF-1 and VEGF levels in older adults.

The relatively low-intensity nature of our exercise program may explain the lack of change in most inflammatory markers, a factor not previously evaluated in exercise-cognitive interventions. Exercise training may reduce pro-inflammatory levels in older adults [13, 14], however these changes may be confounded by concomitant losses in weight or body fat [48]. While our control group exhibited significant, albeit modest, gains in weight (mean change 0.43 kg) and BMI (0.21 kg/m^2^) over the 6-month intervention, the exercise intervention did not improve other measures of body composition. This is not unexpected given our training focused on high-velocity functional exercises using elastic (resistance) bands, body-weight and dumbbells, and challenging balance and mobility activities, to optimise functional performance rather than targeting fat loss and muscle gains. In partial support of our findings, several interventions over 12-weeks to 12-months reported that lower-intensity, elastic-band PRT did not affect various circulating inflammatory markers in institutionalised [49] or community-dwelling older adults, despite small reductions in body fat percentage [50]. Indeed, greater inflammatory responses to exercise are typically observed in adults (healthy and clinical cohorts) with higher intensity PRT (> 60%1RM) [48], while exercise-induced reductions in body fat of up to 10% may facilitate changes in inflammatory markers [51].

Higher levels of Aβ present in the cerebrospinal fluid and circulating plasma may contribute to greater inflammation, and their deposition into senile plaques in the brain is a key aspect of Alzheimer’s disease (AD) pathology [52]. Following our 6-month DT-FPT program, we observed a significant between-group net benefit (11% decrease) in plasma Aβ (1–40) compared to controls. In contrast to our findings, both 12-weeks of multimodal training (low-moderate intensity walking and PRT) and dual-task exercise cognitive training (multimodal performed with arithmetic and motor tasks) both elicited significant within-group increases (38–45%) in plasma Aβ (1–40) [21]. The mechanisms underlying the change in plasma Aβ levels are unclear, but may reflect a greater degradation and clearance of this protein following exercise [12]. However, it remains unclear if (and what magnitude) our changes in plasma Aβ levels are associated with any cognitive benefits or attenuation of risk for neurodegeneration. The response of cognitive function to our intervention did not vary by polymorphism, supporting the limited number of studies that have reported cognitive benefits independent of genotype [20, 53]. Any deleterious impact of the Met allele on cognition may be diminished in late adulthood (i.e., 75 years), due to the independent effects that dementia and age-related disease have on cognition [54]. Furthermore, although the proportion of ε4 carriers in our study (27%) matched population estimates, it is possible that ε4 and Met may exert greater interactive effects with exercise in older adults with cognitive impairment or disease, rather than relatively healthy older adults (e.g., ε4 carriers with AD may be more responsive to exercise) [24].

There are several strengths to this study. This is the first RCT exploring cognitive responses to a dual-task, multi-component functional power-based training program, including an extensive panel of inflammatory and neurological markers. Second, cluster randomisation was employed to avoid the likelihood of intervention contamination within each village, while researcher blinding reduced potential bias. Thirdly, this study was powered for the primary trial objectives (falls) resulting in a larger sample size than is customary for dual-task exercise interventions. However, several limitations must be considered. The modest level of adherence to our DT-FPT program (~ 50%) likely impacted our findings, although we did not observe any differences to our results in the per protocol analyses. Second, while the sample included older adults at increased falls risk they were relatively healthy without significant functional impairment, and so findings cannot be generalised to frail older adults who may not be able to safely undertake the supervised DT-FPT program. Further to this, the use of antihypertensive medication may influence the study outcomes since they can alter risk factors related to cognition (e.g., inflammation, cerebrovascular events) [55]. Exploratory analysis revealed there were significant interactions between baseline antihypertensive medication use and changes in two cognitive outcomes between the groups (interaction: CogState Brief Battery, P = 0.024; psychomotor function-attention composite, P = 0.011). Specifically, in those not taking antihypertensive medications, DT-FPT participants exhibited greater benefits compared to controls for both the CogState Brief Battery [Z-score net difference: 0.23 SD (0.05, 0.41); P = 0.013] and psychomotor function-attention composite [0.41 SD (0.17, 0.65); P = 0.001]. While these findings must be interpreted with caution as this analysis represents unplanned secondary analysis, they highlight that the effectiveness of exercise interventions on cognitive function might be influenced by factors such as medication use which warrants further investigation. Third, there was no adjustment for multiple comparisons but based on the number of tests undertaken we cannot discount that our significant findings may be due to chance. Finally, the sample was predominantly Caucasian and well-educated which may not be representative of the older population.

In conclusion, this 6-month cluster RCT indicates that DT-FPT can provide some cognitive benefits to healthy older adults at increased falls risk which may delay the onset of cognitive decline, however it appears that any improvements were not related to concurrent changes in inflammatory and/or neurological markers. Further research is needed to understand the underlying mechanism(s) by which exercise-cognitive training can improve cognition.

## Supplementary Information

Below is the link to the electronic supplementary material.

Supplementary file1 (DOCX 32.9 KB)
