# Supplementary material for: Effects of a 6-month dual-task, power-based exercise program on cognitive function, neurological and inflammatory markers in older adults: secondary analysis of a cluster randomised controlled trial
Source: GeroScience. 2024 Aug 28;47(1):1251–68. doi: 10.1007/s11357-024-01316-8 (PMC11872956; doi:10.1007/s11357-024-01316-8)
Supplement: Supplementary file 1 — Supplementary file1 (DOCX 32.9 KB) [file 11357_2024_1316_MOESM1_ESM.docx]

# Supplementary Table 1: Cluster size per village, retention of participants at 6-month follow-up testing, number of participants from each village who did not attend 6-month testing in the dual task functional power training (DT-FPT) or usual care control (CON), and average exercise adherence of villages in the intervention group.

| **DT-FPT** | | | |  |  |  |  |  |  | **CON** |  |  |  |
| --- | --- | --- | --- | --- | --- | --- | --- | --- | --- | --- | --- | --- | --- |
|  |  |  | Reasons for participants not attending follow-up | | |  |  |  |  |  | Reasons for participants not attending follow-up | | |
|  | **Baseline** |  |  |  |  |  |  |  | **Baseline** |  |  |  |  |
| **Village** | **cluster** | **Retention** | **WD,** | **Ill / NA,** | **Lost to** | **Mean ± SD** |  | **Village** | **cluster** | **Retention** | **WD,** | **Ill / NA,** | **Lost to** |
| **No.** | **size,** | **n (%)** | **n** | **n** | **follow up, n** | **Adherence (%** |  | **No.** | **size,** | **n (%)** | **n** | **n** | **follow up, n** |
|  | **n** |  |  |  |  |  |  |  | **n** |  |  |  |  |
| 2 | 11 | 10 (91%) | 1 | 0 | 0 | 62.7 ± 28.7 |  | 1 | 12 | 9 (75%) | 1 | 2 | 0 |
| 3 | 12 | 10 (83%) | 2 | 0 | 0 | 61.5 ± 31.2 |  | 5 | 7 | 7 (100%) | 0 | 0 | 0 |
| 4 | 11 | 9 (82%) | 2 | 0 | 0 | 44.9 ± 36.8 |  | 6 | 8 | 5 (63%) | 3 | 0 | 0 |
| 8 | 5 | 5 (100%) | 0 | 0 | 0 | 74.5 ± 13.8 |  | 7 | 16 | 10 (63%) | 4 | 1 | 1 |
| 10 | 10 | 9 (90%) | 0 | 1 | 0 | 48.2 ± 22.4 |  | 9 | 17 | 13 (77%) | 3 | 1 | 0 |
| 13 | 19 | 14 (74%) | 1 | 3 | 1 | 38.2 ± 26.1 |  | 11 | 14 | 13 (93%) | 1 | 0 | 0 |
| 14 | 21 | 12 (57%) | 7 | 1 | 1 | 42.8 ± 37.9 |  | 12 | 23 | 20 (87%) | 3 | 0 | 0 |
| 15 | 15 | 10 (67%) | 4 | 1 | 0 | 61.3 ± 34.1 |  | 16 | 12 | 10 (83%) | 1 | 1 | 0 |
| 17 | 16 | 12 (75%) | 4 | 0 | 0 | 47.8 ± 40.4 |  | 18 | 8 | 8 (100%) | 0 | 0 | 0 |
| 21 | 21 | 14 (67%) | 6 | 1 | 0 | 39.6 ± 27.0 |  | 19 | 15 | 13 (87%) | 0 | 2 | 0 |
| 22 | 15 | 11 (73%) | 4 | 0 | 0 | 59.9 ± 29.3 |  | 20 | 12 | 9 (75%) | 2 | 1 | 0 |
| **Total** | **156** | **116 (74%)** | **31** | **7** | **2** | **50.1 ± 32.3** |  | **Total** | **144** | **117 (81%)** | **18** | **8** | **1** |

WD: Withdrawals, Ill/NA: ill or not available for 6-month testing.

# Supplementary Table 2: Mean baseline characteristics in participants that provided data at both time points, and those not providing data at 6-month follow-up testing, within DT-FPT and CON groups.

| **Characteristics** | **DT-FPT** | | **CON** | |
| --- | --- | --- | --- | --- |
|  | Completed intervention | Did not attend 6-month testing | Completed intervention | Did not attend 6- month testing |
| n | 116 | 39 | 117 | 27 |
| Women, n (%) | 75 (65%) | 26 (67%) | 96 (82%) | 22 (81%) |
| Age, years | 76.7 ± 6.6 | 78.4 ± 6.3 | 77.8 ± 7.0 | 77.4 ± 7.9 |
| Height, cm1 | 164.1 ± 9.2 | 160.8 ± 8.3* | 159.9 ± 7.4 | 160.6 ± 10.6 |
| Weight, kg1 | 78.9 ± 16.6 | 74.2 ± 13.3 | 74.4 ± 13.9 | 73.7 ± 15.1 |
| BMI (kg/m2)1 | 29.2 ± 5.4 | 28.7 ± 4.8 | 29.1 ± 5.0 | 28.5 ± 4.5 |
| Smoking status, n (%)2 |  |  |  |  |
| *Current/ Ex-Smoker* | 56 (48%) | 16 (42%) | 43 (37%) | 13 (48%) |
| *Non-smoker* | 60 (52%) | 22 (58%) | 74 (63%) | 12 (52%) |
| DASS-21-Depression 3 | 2.0 ± 6.0 | 1.0 ± 4.0 | 2.0 ± 6.0 | 2.0 ± 10.0 |
| ApoE-ε4 carrier, n (%)4 | 31 (27%) | 11 (34%) | 29 (25%) | 10 (43%) |
| BDNF Met carrier, n (%)4 | 37 (33%) | 13 (41%) | 34 (30%) | 8 (35%) |
| Presence of chronic health conditions2 | 114 (98%) | 36 (95%) | 112 (96%) | 25 (100%) |
| *No. of conditions in those with a condition* | 3.0 ± 2.0 | 3.0 ± 2.0 | 3.0 ± 2.0 | 3.0 ± 3.0 |
| Presence of cardiometabolic risk factors, n (%)2 | 99 (85%) | 37 (97%) | 97 (83%) | 21 (84%) |

## Supplementary Table 2: continued

| **Characteristics** | **DT-FPT** | | **CON** | |
| --- | --- | --- | --- | --- |
|  | Completed intervention | Did not attend 6- month testing | Completed intervention | Did not attend 6-month testing |
| Employment Status, n (%)2 |  |  |  |  |
| *Retired / Not employed* | 109 (94%) | 36 (95%) | 110 (94%) | 23 (92%) |
| *Part time employment* | 4 (4%) | 0 (0%) | 2 (2%) | 1 (4%) |
| *Home duties / Other* | 3 (2%) | 2 (5%) | 5 (4%) | 1 (4%) |
| Caucasian, n (%)2 | 115 (99%) | 37 (97%) | 114 (97%) | 24 (96%) |
| Education, n (%)2 |  |  |  |  |
| *Primary/Some High School* | 43 (37%) | 14 (37%) | 48 (41%) | 9 (36%) |
| *Completed High School/ Technical Trade Cert.* | 40 (35%) | 10 (26%) | 39 (33%) | 10 (40%) |
| *University/Tertiary level* | 33 (28%) | 14 (37%) | 30 (26%) | 6 (24%) |
| Medications taken2 |  |  |  |  |
| *Antihypertensive, n (%)* | 81 (70%) | 31 (82%) | 84 (72%) | 17 (68%) |
| *Lipid-lowering, n (%)* | 61 (53%) | 21 (55%) | 54 (46%) | 10 (40%) |
| *NSAIDs, n (%)* | 12 (10%) | 6 (16%) | 18 (15%) | 4 (16%) |
| *Anti-depressant, n (%)* | 26 (22%) | 11 (29%) | 25 (21%) | 6 (24%) |
| *Diabetic, n (%)* | 13 (11%) | 3 (8%) | 18 (15%) | 0 (0%) |
| *Neurological, n (%)* | 2 (5%) | 2 (2%) | 3 (3%) | 1 (4%) |
| *Analgesics (non-NSAIDs), n (%)* | 9 (8%) | 8 (21%) | 12 (10%) | 4 (16%) |
| *Sleep aids, n (%)* | 9 (8%) | 2 (5%) | 12 (10%) | 2 (8%) |

Values are mean ± SD except DASS-21-Depression subscale score and Number of health conditions (median ± Interquartile Range). ApoE: apolipoprotein; BDNF: brain derived neurotrophic factor; BMI: body mass index; CON: usual care control group; DASS: Depression, anxiety and stress scale; DT-FPT: dual-task functional power training; NSAID: Nonsteroidal anti-inflammatory drug.

Neurological medication included anti-Parkinson and anti-epileptic medication. 1 Missing data for DT-FPT: n=1 (1%); 2 Missing data for DT-FPT: n=1 (1%), CON: n=2 (1%); 3 Missing data for DT-FPT: n=1 (1%), CON: n=1 (1%); 4 Missing data for DT-FPT: n=10 (6%), CON:

n=7 (5%). *P<0.05 vs ’Completed intervention’.

# Supplementary Table 3: Imputed data: Mean baseline cognitive performance Z-Scores, the within-group changes relative to baseline and net between-group differences for the change after 6 months in the dual-task functional power training (DT-FPT) and control (CON) groups.

|  |  | **DT-FPT** |  | **CON** | **Intervention effects** | |
| --- | --- | --- | --- | --- | --- | --- |
|  | **n** | **Mean ± SD or (95% CI)** | **n** | **Mean ± SD or (95% CI)** | **Net Difference (95% CI)** | **P- values ^1^** |
| **GMT (Executive function)** | | | | | | |
| Baseline | 155 | 0.15 (0.002, 0.30) | 144 | -0.16 (-0.33, 0.01) |  |  |
| ∆ 6 months | 155 | 0.04 (-0.10, 0.18) | 144 | 0.08 (-0.07, 0.24) | -0.05 (-0.25, 0.16) | 0.831 \| 0.745 |
| **DET (Psychomotor function)** | | | | | | |
| Baseline | 155 | 0.01 (-0.15, 0.17) | 144 | -0.01 (-0.17, 0.15) |  |  |
| ∆ 6 months | 155 | -0.19 (-0.36, -0.02)* | 144 | -0.29 (-0.47, -0.12)† | 0.10 (-0.14, 0.35) | 0.232 \| 0.120 |
| **IDN (Attention/Choice reaction time)** | | | | | | |
| Baseline | 155 | -0.02 (-0.18, 0.14) | 144 | 0.02 (-0.14, 0.18) |  |  |
| ∆ 6 months | 155 | 0.07 (-0.07, 0.21) | 144 | -0.06 (-0.20, 0.09) | 0.13 (-0.07, 0.33) | 0.168 \| 0.122 |
| **OCL (Visual learning)** | | | | | | |
| Baseline | 155 | 0.08 (-0.08, 0.24) | 144 | -0.08 (-0.25, 0.08) |  |  |
| ∆ 6 months | 155 | 0.09 (-0.08, 0.26) | 144 | 0.15 (-0.03, 0.33) | -0.06 (-0.31, 0.19) | 0.898 \| 0.432 |
| **ONB (Working memory)** | | | | | | |
| Baseline | 155 | 0.08 (-0.09, 0.25) | 144 | -0.09 (-0.24, 0.07) |  |  |
| ∆ 6 months | 155 | 0.06 (-0.05, 0.18) | 144 | 0.12 (-0.003, 0.24) | -0.06 (-0.22, 0.11) | 0.840 \| 0.779 |

**Supplementary Table 3: continued**

|  |  | **DT-FPT** |  | **CON** | **Intervention effects** | |
| --- | --- | --- | --- | --- | --- | --- |
|  | **n** | **Mean ± SD or (95% CI)** | **n** | **Mean ± SD or (95% CI)** | **Net Difference (95% CI)** | **P- values ^1^** |
| **Global cognitive function** | | | | | | |
| Baseline | 155 | 0.06 (-0.05, 0.17) | 144 | -0.06 (-0.17, 0.04) |  |  |
| ∆ 6 months | 155 | 0.01 (-0.06, 0.09) | 144 | 0.001 (-0.07, 0.07) | 0.01 (-0.09, 0.12) | 0.450 \| 0.254 |
| **Learning-Working Memory** | | | | | | |
| Baseline | 155 | 0.08 (-0.05, 0.21) | 144 | -0.08 (-0.20, 0.03) |  |  |
| ∆ 6 months | 155 | 0.08 (-0.03, 0.18) | 144 | 0.14 (0.04, 0.24)** | -0.06 (-0.21, 0.09) | 0.745 \| 0.893 |
| **Psychomotor function-Attention** | | | | | | |
| Baseline | 155 | -0.004 (-0.15, 0.14) | 144 | 0.005 (-0.14, 0.15) |  |  |
| ∆ 6 months | 155 | -0.06 (-0.18, 0.06) | 144 | -0.18 (-0.31, -0.04)** | 0.12 (-0.06, 0.30) | 0.144 \| 0.075 |
| **CogState Brief Battery** | | | | | | |
| Baseline | 155 | 0.04 (-0.08, 0.16) | 144 | -0.04 (-0.15, 0.07) |  |  |
| ∆ 6 months | 155 | 0.01 (-0.08, 0.09) | 144 | -0.02 (-0.10, 0.06) | 0.03 (-0.09, 0.15) | 0.352 \| 0.161 |

Baseline values are means with 95% CI; within and between-group (net) differences are unadjusted means with 95% CI. Net differences (95% CIs) were calculated by subtracting within-group changes from baseline for the CON group from within-group changes for the DT-FPT group after 6 months. 1 P-values for time and group-by-time interaction terms were derived from linear mixed models and represent Model 1 (unadjusted) and Model 2 (adjusted for age, sex, education level, and baseline values). DET: Detection task; GMT: Groton Maze Learning Test; IDN: Identification task; OCL: One Card Learning task; ONB: One Back task. * P<0.05, ** P<0.01, † P≤0.001, within-group change relative to baseline.

# Supplementary Table 4: Imputed data: Mean baseline inflammatory profile, the within-group changes relative to baseline and net between-group differences for the change after 6 months in the dual-task functional power training (DT-FPT) and control (CON) groups.

|  |  | **DT-FPT** |  | **CON** | **Intervention effects** | |
| --- | --- | --- | --- | --- | --- | --- |
|  | **n** | **Mean ± SD or (95% CI)** | **n** | **Mean ± SD or (95% CI)** | **Net Difference (95% CI)** | **P- values ^1^** |
| **IL-6** |  |  |  |  |  |  |
| Baseline | 155 | 1.90 (1.61, 2.20) | 144 | 2.92 (2.35, 3.50) |  |  |
| % ∆6 months | 155 | -0.2 (-14.2, 13.7) | 144 | -10.6 (-26.7, 5.5) | 10.4 (-11.0, 31.8) | 0.866 \| 0.856 |
| **TNF-α** |  |  |  |  |  |  |
| Baseline | 155 | 10.2 (9.2, 11.2) | 144 | 12.0 (10.6, 13.4) |  |  |
| % ∆6 months | 155 | -7.5 (-14.6, -0.3)* | 144 | -3.6 (-12.0, 4.8) | -3.9 (-14.8, 7.0) | 0.385 \| 0.344 |
| **IL-1β** |  |  |  |  |  |  |
| Baseline | 155 | 1.52 (0.9, 2.2) | 144 | 1.87 (1.3, 2.4) |  |  |
| % ∆6 months | 155 | -2.3 (-17.8, 13.1) | 144 | -3.7 (-19.4, 12.1) | 1.3 (-20.6, 23.3) | 0.617 \| 0.715 |
| **IL-8** |  |  |  |  |  |  |
| Baseline | 155 | 14.3 (12.7, 15.9) | 144 | 16.5 (14.0, 18.9) |  |  |
| % ∆6 months | 155 | -5.4 (-11.5, 0.6) | 144 | -9.2 (-15.3, -3.1)** | 3.8 (-5.0, 12.7) | 0.608 \| 0.703 |
| **CRP** |  |  |  |  |  |  |
| Baseline | 155 | 2.78 (2.21, 3.35) | 144 | 3.36 (2.74, 3.99) |  |  |
| % ∆6 months | 155 | 2.4 (-18.0, 22.7) | 144 | -4.7 (-23.7, 14.4) | 7.0 (-21.0, 35.0) | 0.844 \| 0.623 |

**Supplementary Table 4: continued**

|  |  | **DT-FPT** |  | **CON** | **Intervention effects** | |
| --- | --- | --- | --- | --- | --- | --- |
|  | **n** | **Mean ± SD or (95% CI)** | **n** | **Mean ± SD or (95% CI)** | **Net Difference (95% CI)** | **P- values ^1^** |
| **IL-10** |  |  |  |  |  |  |
| Baseline | 155 | 4.68 (3.29, 6.07) | 144 | 8.64 (6.00, 11.3) |  |  |
| % ∆6 months | 155 | -13.0 (-32.1, 6.1) | 144 | -19.6 (-37.0, -2.3)* | 6.6 (-18.9, 32.1) | 0.974 \| 0.986 |
| **IL-4** |  |  |  |  |  |  |
| Baseline | 155 | 18.8 (12.5, 25.0) | 144 | 30.8 (20.4, 41.2) |  |  |
| % ∆6 months | 155 | 18.1 (-3.2, 39.4) | 144 | -6.7 (-27.2, 13.9) | 24.7 (-3.7, 53.2) | 0.854 **\|** 0.845 |
| **Pro-inflammatory Z-Score** | | | | | | |
| Baseline | 155 | -0.10 (-0.19, -0.02) | 144 | 0.11 (-0.01, 0.23) |  |  |
| ∆6 months | 155 | 0.03 (-0.10, 0.17) | 144 | -0.09 (-0.18, 0.002) | 0.12 (-0.04, 0.29) | 0.635 \| 0.549 |
| **Anti-inflammatory Z-Score** | | | | | | |
| Baseline | 155 | -0.13 (-0.23, -0.03) | 144 | 0.14 (-0.05, 0.33) |  |  |
| ∆6 months | 155 | 0.01 (-0.04, 0.05) | 144 | -0.02 (-0.08, 0.05) | 0.02 (-0.06, 0.10) | 0.785 \| 0.633 |
| **Single composite inflammatory Z-score** | | | | | | |
| Baseline | 155 | -0.11 (-0.19, -0.03) | 144 | 0.12 (-0.01, 0.24) |  |  |
| ∆6 months | 155 | 0.02 (-0.07, 0.12) | 144 | -0.06 (-0.13, 0.001) | 0.09 (-0.03, 0.20) | 0.818 \| 0.733 |

Baseline values are means ± SD; within and between-group (net) differences are means with 95% confidence intervals (CI), and calculated by subtracting within-group changes from baseline for the CON group from within-group changes for the DT-FPT group after 6 months. Percentages are calculated as log-transformed within-group and between-group changes x 100. Inflammatory Z-scores and changes are presented as raw data, analysis was conducted on Z scores constructed from log-transformed concentrations. 1 P-values were derived from linear mixed models and represent Model 1 (unadjusted) and Model 2 (adjusted for baseline, age, sex, BMI). Cytokines, concentrations presented in pg/mL, CRP measured in mg/L. CRP: C-reactive protein; IL-1β: interleukin-1 beta; IL-4: interleukin-4; IL-6: interleukin-6; IL-8: interleukin-8; IL-10: interleukin-10; TNF-α: tumor- necrosis factor alpha.

# Supplementary Table 5: Imputed data: Mean baseline neurological concentrations, the within-group changes relative to baseline and net between-group differences for the change after 6 months in the dual-task functional power training (DT-FPT) and control (CON) groups.

|  |  | **DT-FPT** |  | **CON** | **Intervention effects** | |
| --- | --- | --- | --- | --- | --- | --- |
|  | **n** | **Mean ± SD or (95% CI)** | **n** | **Mean ± SD or (95% CI)** | **Net Difference (95% CI)** | **P- values ^1^** |
| **Aβ (1-40)** |  |  |  |  |  |  |
| Baseline | 155 | 113.3 (105.3, 121.3) | 144 | 109.4 (103.7, 115.0) |  |  |
| % ∆6 months | 155 | 0.02 (-5.2, 5.2) | 144 | 7.5 (2.2, 12.7)** | -7.4 (-14.7, -0.2) | 0.096 \| 0.118 |
| **Aβ (1-42)** |  |  |  |  |  |  |
| Baseline | 155 | 5.62 (4.52, 6.73) | 144 | 4.21 (3.37, 5.06) |  |  |
| % ∆6 months | 155 | 20.5 (3.1, 38.0)* | 144 | 15.8 (-3.4, 35.1) | 4.7 (-21.0, 30.4) | 0.477 \| 0.410 |
| **BDNF** |  |  |  |  |  |  |
| Baseline | 155 | 32.3 (30.8, 33.9) | 144 | 29.0 (27.4, 30.6) |  |  |
| % ∆6 months | 155 | 1.5 (-4.3, 7.2) | 144 | 9.7 (-0.003, 19.4) | -8.2 (-19.1, 2.7) | 0.631 \| 0.510 |
| **IGF-1** |  |  |  |  |  |  |
| Baseline | 155 | 16.8 (16.0, 17.7) | 144 | 16.6 (15.8, 17.5) |  |  |
| % ∆6 months | 155 | 1.1 (-2.6, 4.7) | 144 | -0.12 (-3.6, 3.4) | 1.2 (-3.6, 6.0) | 0.654 \| 0.720 |
| **VEGF** |  |  |  |  |  |  |
| Baseline | 155 | 346 (303, 389) | 144 | 410 (345, 475) |  |  |
| % ∆6 months | 155 | -6.2 (-16.1, 3.6) | 144 | -9.0 (-19.8, 1.9) | 2.7 (-11.1, 16.6) | 0.842 \| 0.599 |

Baseline values are means ± SD; within and between-group (net) differences are means with 95% confidence intervals (CI), and calculated by subtracting within-group changes from baseline for the CON group from within-group changes for the DT-FPT group after 6 months. Percentages are calculated as log- transformed within-group and between-group changes x 100, except for BDNF and IGF-1 which were not transformed. 1 P-values were derived from linear mixed models and represent Model 1 (unadjusted) and Model 2 (adjusted for baseline, age, sex, BMI). Aβ and VEGF concentrations presented in pg/mL, BDNF as ng/mL and IGF as nmol/L. Aβ: amyloid beta; BDNF: brain derived neurotrophic factor; IGF-1: insulin-like growth factor-1; VEGF: vascular endothelial growth factor.

# Supplementary Table 6: Mean baseline body composition variables, within-group changes relative to baseline and net between-group differences for the change after 6 months in the dual-task functional power training (DT-FPT) and control (CON) groups.

|  |  | **DT-FPT** |  | **CON** | **Intervention effects** | |
| --- | --- | --- | --- | --- | --- | --- |
|  | **n** | **Mean ± SD or (95% CI)** | **n** | **Mean ± SD or (95% CI)** | **Net Difference (95% CI)** | **P- values ^1^** |
| **Weight** |  |  |  |  |  |  |
| Baseline, kg | 154 | 77.7 ± 16.0 | 144 | 74.3 ± 14.1 |  |  |
| ∆ 6 months | 116 | -0.24 (-0.66, 0.19) | 117 | 0.43 (0.05, 0.80)* | -0.67 (-1.23, -0.10) | 0.056 \| 0.089 |
| **BMI** |  |  |  |  |  |  |
| Baseline, kg/m2 | 154 | 29.1 ± 5.2 | 144 | 29.0 ± 4.9 |  |  |
| ∆ 6 months | 116 | -0.06 (-0.21, 0.09) | 117 | 0.21 (0.05, 0.36)* | -0.26 (-0.48, -0.05) | **0.036** \| 0.069 |
| **Fat mass** |  |  |  |  |  |  |
| Baseline, kg | 148 | 29.3 ± 10.6 | 139 | 29.5 ± 8.9 |  |  |
| ∆ 6 months | 110 | 0.18 (-0.48, 0.83) | 111 | 0.37 (-0.01, 0.74) | -0.19 (-0.94, 0.55) | 0.706 \| 0.924 |
| **Total body fat** |  |  |  |  |  |  |
| Baseline, % | 148 | 36.9 ± 8.2 | 139 | 39.1 ± 6.7 |  |  |
| % ∆ 6 months | 110 | 0.37 (-0.24, 0.99) | 111 | 0.21 (-0.22, 0.64) | 0.17 (-0.58, 0.91) | 0.944 \| 0.658 |
| **Fat-free mass** |  |  |  |  |  |  |
| Baseline, kg | 148 | 48.8 ± 9.8 | 139 | 45.0 ± 8.3 |  |  |
| ∆ 6 months | 110 | -0.42 (-0.89, 0.06) | 111 | -0.001 (-0.34, 0.34) | -0.42 (-1.00, 0.16) | 0.495 \| 0.390 |

Baseline values are means ± SD; within and between-group (net) differences are unadjusted means of absolute and percentage change with 95% CI. Net differences (95% CIs) were calculated by subtracting within-group percentage changes from baseline for the CON group from within-group percentage changes for the DT-FPT group after 6 months. Missing data points at 6-month testing were due to injury (DT-FPT, n=2; CON, n=1) or the presence of a pacemaker (DT- FPT, n=4; CON, n=5). 1 P-values for time and group-by-time interaction terms were derived from linear mixed models and represent model 1 (adjusting for sex), and Model 2 (adjusted for baseline, sex). BMI: body mass index. * P=0.05, ** P<0.01.

9
